# BDNF Expression in Larval and Adult Zebrafish Brain: Distribution and Cell Identification

**DOI:** 10.1371/journal.pone.0158057

**Published:** 2016-06-23

**Authors:** Pietro Cacialli, Marie-Madeleine Gueguen, Pascal Coumailleau, Livia D’Angelo, Olivier Kah, Carla Lucini, Elisabeth Pellegrini

**Affiliations:** 1 INSERM U1085, Research Institute in Health, Environment and Occupation (IRSET), University of Rennes 1, Rennes, France; 2 Department of Veterinary Medicine and Animal Productions, University of Naples Federico II, Napoli, Italy; University of Rouen, France, FRANCE

## Abstract

Brain-derived neurotrophic factor (BDNF), a member of the neurotrophin family, has emerged as an active mediator in many essential functions in the central nervous system of mammals. BDNF plays significant roles in neurogenesis, neuronal maturation and/or synaptic plasticity and is involved in cognitive functions such as learning and memory. Despite the vast literature present in mammals, studies devoted to BDNF in the brain of other animal models are scarse. Zebrafish is a teleost fish widely known for developmental genetic studies and is emerging as model for translational neuroscience research. In addition, its brain shows many sites of adult neurogenesis allowing higher regenerative properties after traumatic injuries. To add further knowledge on neurotrophic factors in vertebrate brain models, we decided to determine the distribution of *bdnf* mRNAs in the larval and adult zebrafish brain and to characterize the phenotype of cells expressing *bdnf* mRNAs by means of double staining studies. Our results showed that *bdnf* mRNAs were widely expressed in the brain of 7 days old larvae and throughout the whole brain of mature female and male zebrafish. In adults, *bdnf* mRNAs were mainly observed in the dorsal telencephalon, preoptic area, dorsal thalamus, posterior tuberculum, hypothalamus, synencephalon, optic tectum and medulla oblongata. By combining immunohistochemistry with in situ hybridization, we showed that *bdnf* mRNAs were never expressed by radial glial cells or proliferating cells. By contrast, *bdnf* transcripts were expressed in cells with neuronal phenotype in all brain regions investigated. Our results provide the first demonstration that the brain of zebrafish expresses *bdnf* mRNAs in neurons and open new fields of research on the role of the BDNF factor in brain mechanisms in normal and brain repairs situations.

## Introduction

Brain derived neurotrophic factor (BDNF) is a protein that belongs to the neurotrophin family, including nerve growth factor (NGF) and neurotrophin (NT) 3, NT 4/5 and NT 6/7 [1]. All neurotrophins interact with two types of receptors, tropomyosin-related receptor kinase (Trk) and p75 neurotrophin receptor (p75NTR). In the brain of mammals, BDNF promotes through TrkB receptor, neuronal survival, growth, differentiation and synaptic plasticity [2]. In addition, BDNF has been shown to modulate, through p75NTR receptor, neuronal migration [3], myelination [4] and neuronal apoptosis [5, 6]. Furthermore, BDNF seems to be involved in modulating memory, learning processes [7], age-dependent alterations of hippocampus [8, 9] and neurodegenerative diseases [10–12]. The *bdnf* gene is composed of multiple alternative exons: 10 in human, 8 in mouse, and 6 in non-mammalian vertebrates [13]. Multiple promoters can modulate the tissue specific transcription of the *bdnf* gene [14–17]. This gene is well conserved across vertebrate evolution [14, 18, 19] and some regulatory sequences in the 5' UTR of the *bdnf* gene appear highly conserved between zebrafish and mammals [18], suggesting conserved functions.

*Bdnf* mRNAs and protein distributions have been described in the brain of different vertebrates species during development and in adults. In rat, *bdnf* transcripts dramatically increased between embryonic days 11 and 12, a timing coinciding with neurogenesis [20]. Studies performed in rat, mouse and pig showed that *bdnf* transcripts and proteins were detected at different postnatal stages (newborn, adult) in regions such as hippocampus, hypothalamus, cerebral cortex, amygdala and brain stem adrenergic nuclei [20–27]. In the human brain, *bdnf* mRNAs were also reported in the hippocampus, the amygdala and the septum [28, 29]. There are few studies in birds and amphibians, where BDNF has been described. In songbirds, *bdnf* is expressed in brain nuclei involved in sensorimotor integration of song learning [30–32]. In amphibians, *bdnf* mRNAs and immunoreactivity were described in optic tectum and hypothalamus [33, 34]. In adult teleost fishes, *bdnf* mRNAs are reported in the brain of eel [35], perciform (*Cichlasoma dimerus)* [36] and Turquoise killifish (*Nothobranchius furzeri)* [37]. In zebrafish, *bdnf* mRNAs are present early during embryonic development and whole-mount in situ hybridization experiments at early developmental stages demonstrated the presence of *bdnf* transcripts in the forebrain, midbrain and hindbrain [38].

A wealth of data obtained from rodent studies points to a role for BDNF in neurogenesis. BDNF contributes to the regulation of neurogenesis during development by promoting neuronal survival and differentiation [39, 40], but also plays roles in adult neurogenesis. However, BDNF effects in adult neurogenesis are not completely established and studies have reported conflicting results. In some studies, BDNF infusion into the ventricle of adult rat substantially increased the number of newly formed cells in many regions, the majority of which differentiate into neurons [41, 42]. BDNF administration in the hippocampus was associated with an increased neurogenesis of granule cells in the dentate gyrus [43]. However, more recently, Galvao and colleagues obtained contrasting results suggesting that BDNF delivered intraventricularly in mice and rats failed to enhance subventricular zone neurogenesis and even reduced it [44]. Transgenic mouse models with *bdnf* gene expression or signalling disruption have been produced to decipher the role of BDNF in regulating neural stem cells but again a clear role of BDNF cannot be stated. Mutant mice lacking BDNF did not survive and had severe neuronal deficits [45, 46]. In heterozygous BDNF knockout mice, proliferation of neural stem cells was decreased in the dentate gyrus of the hippocampus [47]. In contrast, conditional knockout mice with depletion of BDNF in mature neurons exhibited an increase in hippocampal proliferation [48]. The role of BDNF in brain regenerative processes was also investigated in rodents. After experimentally induced brain injury, the expression of *bdnf* was up-regulated close to the lesioned area, probably as part of the repair mechanism following trauma [49–51]. In the mouse, BDNF treatment improved long-term survival and maturation of newly formed cells derived from the subcallosal zone after cortex injury [52]. Conversely, in transgenic mice in which *bdnf* gene expression was abolished in astrocytes, a low number of new generated oligodendrocytes and consequently a larger damaged area were observed after hypoperfusion of the carotid artery [53]. All together, those reports on the role of BDNF in neurogenesis generated conflicting results, which need deeper investigations.

Over the last ten years zebrafish has become an interesting model organism to study the molecular and cell biology of the vertebrate brain [54, 55] and has emerged as a model species for translational research in various neuroscience areas, such as depressive disorders [56], neurodegeneration [57], motor neuron disease [58], autism spectrum disorders [59] and Alzheimer’s disease [60]. In addition, compared to mammals, the brain of adult zebrafish exhibits a high number of proliferative areas distributed along the ventricles of the telencephalon, diencephalon and mesencephalon [61–65], a feature tightly linked to the persistence of radial glial progenitors [61]. Moreover, as shown by several studies, teleost fishes, including zebrafish, exhibit an outstanding capacity to regenerate after brain injury [66–69]. The factors supporting this intense neurogenic activity in normal physiological and reparative conditions are not identified to date.

Virtually nothing is known on BDNF functions in the central nervous system of fish. Given the potential role of BDNF in neurogenesis mentioned above and the strong proliferative activity in the brain of adult zebrafish, we have investigated the potential link between this neurotrophin and neurogenesis in zebrafish. First, we studied in detail the distribution of *bdnf* mRNAs in the brain of zebrafish and determined, by means of double staining studies, the phenotype (proliferating cells, glial or neuronal cells) of *bdnf*-expressing cells.

## Materials and Methods

### Animals and tissue processing

Animals were handled and sacrificed in agreement with the guidelines for the use and care of laboratory animals and in compliance with French and European regulations on animal welfare. Animals used in this study were housed in our zebrafish facilities (INRA LPGP, BIOSIT, Rennes, France, agreement number: B 35-238-6) under standard conditions of photoperiod (14 hours light and 10 hours dark) and temperature (28°C). This project was approved by the local animal care and ethics committee (Comité Rennais d'Ethique en matière d'Expérimentation Animale, Rennes, France), under the number EEA B-35-040. Zebrafish did not receive medical treatment prior or during the experience. No deaths occurred in the facilities before the sacrifice of animals used for in situ hybridization and immunohistochemistry experiments. For studies on larvae, adults were spawned, embryos were collected (n = 20) and kept for 7 days in an incubator in 100 ml glass bottles before being sacrificed in ice water and fixed overnight 4°C in phosphate-buffered saline (PBS; pH 7.4) containing 4% paraformaldehyde (PAF). For studies in adults, four months old females (n = 6) and males (n = 6) were sacrificed by overdose of tricaine methanesulfonate (MS-222, 300mg/l). The sex was then determined by direct examination of the gonads under a binocular stereoscopic microscope. After skull opening, the brain was removed and fixed overnight in PBS-PAF. Both larvae and adult brains were processed for paraffin embedding and microtome sections were mounted on poly-lysine slides.

### In situ hybridization

*Bdnf* expression (ie *bdnf* mRNAs presence in cells) was investigated using in situ hybridization. For *bdnf* riboprobes synthesis, we used a pCMV-Sport 6.1 plasmid containing the full-length BDNF cDNA (Unigene DR.132862; Entrez Gene 58118). Antisense and sense riboprobes were generated with DIG RNA Labelling Mix (Roche Diagnostic, Indianapolis, US) by in vitro transcription, using T7 polymerase (Roche-Diagnostic) and SP6 polymerase (Roche-Diagnostic) on plasmid linearized by EcoRI and Not1. To check the specificity of the staining, the sense and antisense riboprobes were always hybridized on adjacent sections. No staining was observed on sections hybridized with the sense riboprobe either in larvae and adults (S1 and S2 Figs) as demonstrated in a previous study in zebrafish [38]. Paraffin sections (7 μm) were deparaffinized with OTTIX and rehydrated through a series of graded ethanol (100–30%). Sections were washed in PBS-NaCl (0.85%) and post-fixed for 20 minutes in PBS-PFA 4%. Tissues were rinsed in PBS and treated for 7 minutes with proteinase K (2 mg/ml) diluted in PBS at 37°C. The reaction was stopped in PBS before post-fixation for 20 minutes in 4% PBS-PAF and the slides were rinsed 10 minutes in PBS and 10 minutes in standard saline citrate (SSC 2x). The sections were incubated overnight at 62.5°C in a moist chamber with the probes (1.5 μg/ml) diluted in hybridization buffer (formamide 50%; SSC 2X, Denhart 5X, yeast tRNA 50 μ/ml, EDTA 4 mM, dextran sulfate 2.5%). On the following day, slides were rinsed with SCC 2x, SCC 2x/formamide 50%, SSC 0.2x and SSC 0.1x. Next, they were dipped in Tris-HCl/NaCl buffer (100 mM, Tris-HCl pH 7.5, 150 mM NaCl) and washed in the same buffer containing 0.1% Triton and 0.5% of milk powder. Sections were incubated overnight at room temperature with anti-digoxigenin alkaline phosphatase Fab fragments (1:2000, Roche Diagnostic). On the next day, slides were rinsed in Tris-HCl/NaCl buffer and washed three times with Tris-HCl 100 mM (pH 8) containing NaCl (100 mM) and MgCl2 (10mM). The hybridization signal was revealed with the HNPP/Fast-Red detection kit (Roche Diagnostic) for 6 or 12 hours according to the manufacturer’s instructions. Sections were washed several times in PBS, mounted in Vectashield medium containing DAPI and coverslipped for microscopic analysis.

### Immunohistochemistry

To get further insights in the identification of *bdnf*-expressing cells (ie cells that express *bdnf* mRNAs), sections were processed for immunohistochemistry just after in situ hybridization. To stop in situ hybridization reaction, sections were dipped 5 minutes in PBS-PAF 4%, washed in PBS and PBS/Triton (0.2%) to perform immunohistochemistry as follows: sections were incubated overnight at room temperature with different primary antibodies diluted in PBS containing 0.5% milk powder. Sections were washed three times in PBS-Triton 0.2% and incubated with goat anti-rabbit or anti-mouse Alexa Fluor 488 (1:200, Invitrogen, ThermoFisher Scientific, France) for 2 hours. Tissue sections were washed in PBS-Triton 0,2%, and slides were mounted with the Vectashield medium containing DAPI for nuclei conterstaining (Vector Laboratories, Burlingame, CA).

Radial glial cells were identified with a rabbit anti-zebrafish aromatase B (this antibody was raised in our laboratory, 1:200) and with a rabbit anti-mouse BLBP (Brain Lipid Binding Protein, 1:100, Chemicon, Temecula, CA, Cat. No. AB9558). The specificity of these antibodies was previously demonstrated in zebrafish [70, 71].

Three neuronal markers were used to identify post-mitotic neurons. The labeling was performed with a mouse monoclonal anti-acetylated-tubulin (1:100, Sigma-Aldrich, France, clone 6-11B-1, Cat. no. T 6793), with a mouse monoclonal anti-HuC/D (1:20, Invitrogen, ThermoFisher Scientific, France, clone 16A11, Cat. no. A21271) or with a mouse monoclonal anti-MAP2 (Microtube-Associated Protein2, 1:100, Abcam, France, clone AP-20, Cat. no. 11268). The specificity of these antibodies was previously confirmed in zebrafish [61, 72].

Proliferative cells were visualized with a monoclonal antibody raised against PCNA (Proliferating Cell Nuclear Antigen, 1:100, Dako, France, clone PC10, Cat. no. M0879). The specificity of this antibody for PCNA has been validated in many vertebrate species including zebrafish [61, 73].

### Microscopy

Sections were observed with an epifluorescence microscope (Olympus Provis, equipped with a DP71 digital camera), an epifluorescence Zeiss (Imager Z1, equipped with the Apotome module) or a confocal microscope Leica SP2. Images were processed with either the Olympus (Cell^F^) Zeiss (AxioVision4) or Leica (LCS Lite) software. Micrographs were acquired in TIFF format and adjusted for light and contrast before being assembled on plates using Photoshop CS4. The nomenclature for brain nuclei in adults and developing zebrafish were taken from Wullimann et al, and Mueller and Wullimann, respectively [74, 75].

## Results

### *Bdnf* mRNAs in the brain of 7 days old larvae

**Fig 1:**
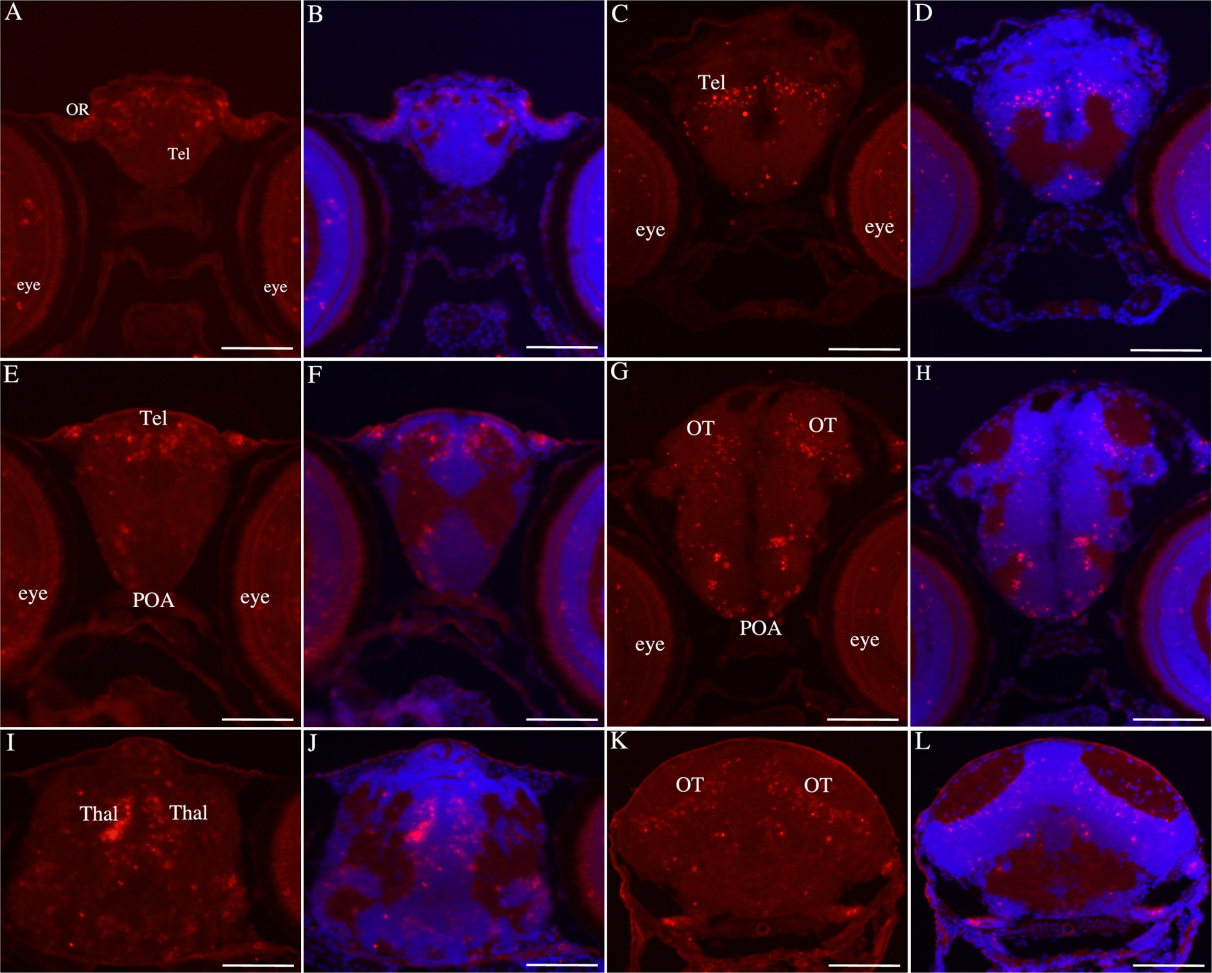
*Bdnf* mRNA are expressed in the brain of 7 days old zebrafish. Olfactory rosettes (**A-B**), telencephalon (**A-F**), preoptic area (**E-H**), dorsal thalamus, (**I-J**), optic tectum (**G-H and K-L**). Figures B, D, F, H, J and L show cell nuclei labeled with DAPI. OR: olfactory rosettes; POA: preoptic area; Tel: telencephalon; Thal: thalamus; OT: optic tectum. Scale bar: 120 μm.

**Fig 2:**
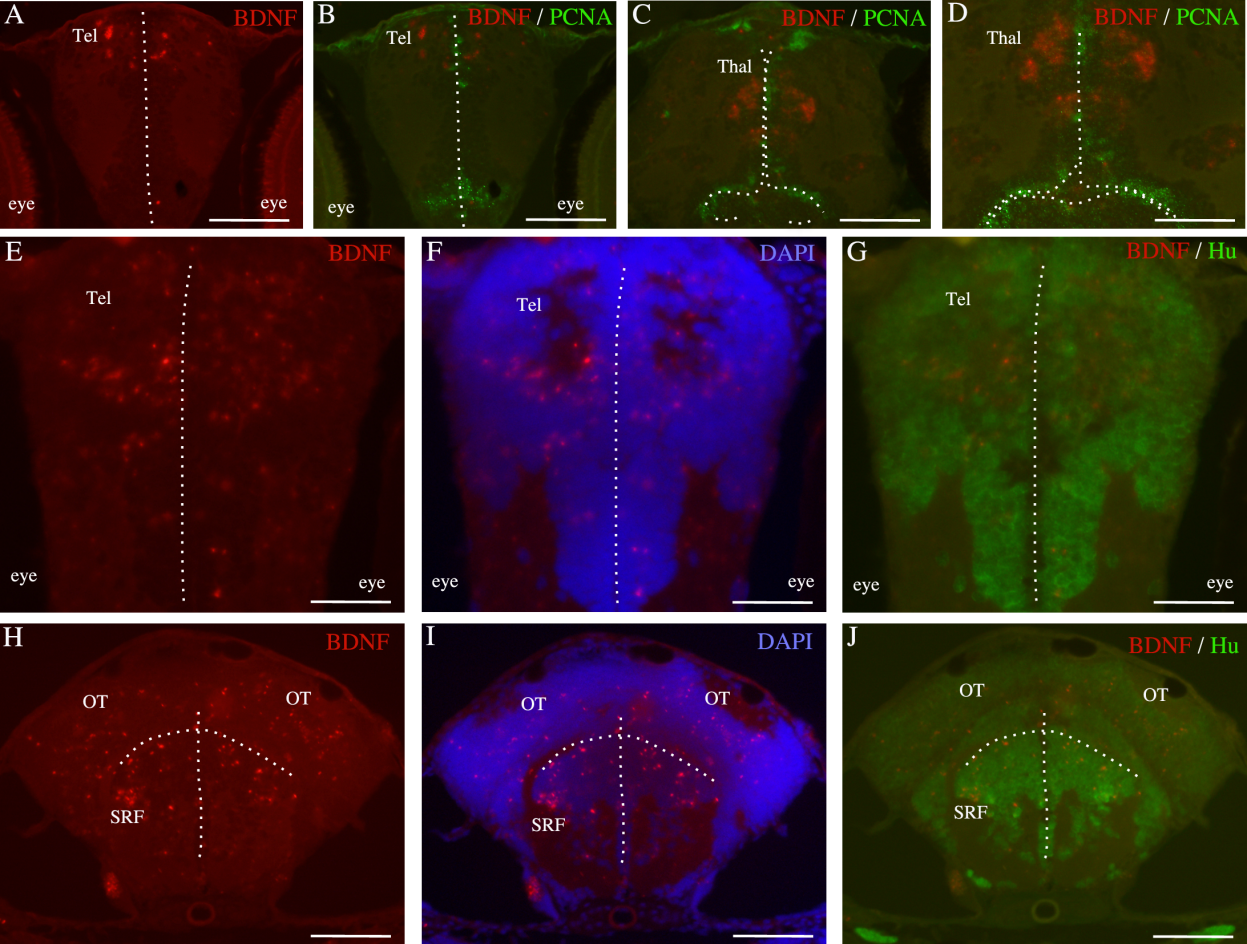
Immunohistochemical characterization of *bdnf*-expressing cells in the brain of 7 days old zebrafish larvae. Double staining for *bdnf* mRNA (red) and PCNA protein (green) on cross-sections through the telencephalon (**A-B**) and the thalamus (**C-D**). Double staining for *bdnf* mRNA (red) and the neuronal marker Hu (green) in the thalamus (**E,F and G**), the optic tectum (**H, I and J**) and at the level of the superior reticular formation (**H, I and J**). In F and I cells nuclei are counterstained with DAPI. The dotted lines indicate the ventricles. OT: optic tectum; SRF: superior reticular formation; Tel: telencephalon; Thal: thalamus. Scale bar: 150 μm in H, I and J. 120 μm in A, B, C. 60 μm in D, E, F and G.

We first studied the presence of *bdnf* mRNAs at early developmental stages. To analyze in detail the distribution of *bdnf* transcripts in the brain of 7 days old larva, we performed in situ hybridization on series of transverse sections of the whole zebrafish brain. The *bdnf* sense riboprobe did not generate any signal (S1 Fig, [38]), confirming the specificity of the labeling obtained with the antisense riboprobe. The antisense riboprobe generated a positive labeling in several brain regions, demonstrating the presence of *bdnf* mRNAs. The Table 1 and Fig 1 summarize the results. From anterior to posterior, *bdnf* mRNAs were detected in the olfactory rosettes and in the anterior telencephalon (Fig 1A–1D). At this level, positive cells were mostly located in the dorsal and medial parts of the dorsal telencephalon. More caudally, labeled cells were still observed in the dorsal telencephalon, preferentially in the central telencephalon and also in the preoptic area (Fig 1E–1H). *Bdnf* mRNAs were particularly abundant in the thalamic area (Fig 1I and 1J) and were also consistently observed in the optic tectum, particularly in the periventricular layer (Fig 1G and 1H–1K and 1L). *Bdnf* mRNAs were also detected at different levels of the midbrain tegmentum, in particular in the reticular formation (Fig 2H–2J).

**Table 1:** *Bdnf* mRNAs in juvenile zebrafish. + = few cells; ++ = moderate number of cells; +++ = numerous cells.

**Olfactofy bulb**	+++
**Pallium**	++
**Subpallium**	+
**Preoptic region**	++
**Habenula**	+
**Dorsal thalamus**	+++
**Dorsal part of posterior tuberculum**	+++
**Ventral part of posterior tuberculum**	++
**Intermediate hypothalamus**	++
**Rostral hypothalamus**	++
**Caudal hypothalamus**	+++
**Optic tectum**	+++
**Tegmentum**	+++

Attempts to identify the phenotype of these *bdnf*-expressing cells were made by using double staining with either PCNA, aromatase B or HuC/D as markers of cell proliferation, radial glial cells or neurons respectively [61, 76]. Data showed that, in each studied region, cells expressing *bdnf* mRNAs never corresponded to PCNA-positive cells indicating that *bdnf*-positive cells are post-mitotic (Fig 2A–2D). *Bdnf*-expressing cells did not co-express aromatase B attesting that they were not radial glia progenitors (data not shown). Regarding the neuronal marker HuC/D, although the coupling of immunohistochemistry with in situ hybridization resulted in some loss of *bdnf* transcripts, it appeared that in many regions such as the telencephalon, the optic tectum and the midbrain tegmentum, the signals generated overlapped, suggesting that *bdnf*-expressing cells may rapidly gained at least a neuronal phenotype (Fig 2E–2J).

### *Bdnf* mRNAs in the brain of adult zebrafish

**Fig 3:**
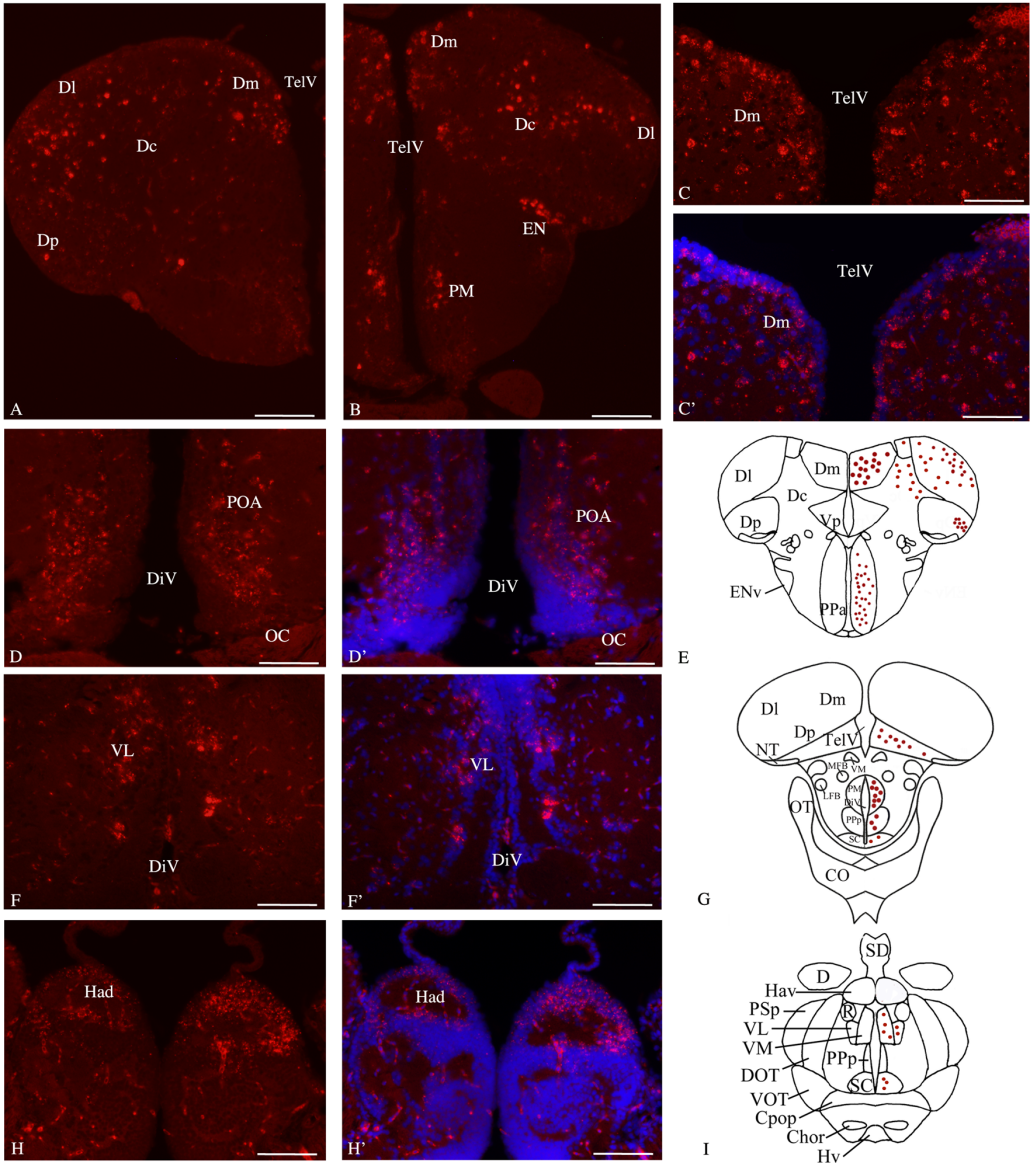
*Bdnf* mRNA distribution in cross-sections through the adult zebrafish forebrain. Telencephalon (**A, B, C, C’ and E**), preoptic area (**B, D, D’ and E, G**), thalamus (**F, F’ and I**) and dorsal habenula (**H, H’**). In C', D', F', H' cell nuclei are labeled in blue with DAPI. E, G and I are representative sections taken from the zebrafish atlas (Wullimann et al., 1996). *Bdnf*-expressing cells are represented by red dots. Dc: central zone of the dorsal telencephalon; DiV: diencephalic ventricle; Dl: lateral zone of the dorsal telencephalon; Dm: medial zone of the dorsal telencephalon; Dp: posterior zone of the dorsal telencephalon; EN: endopedoncular nucleus; Had: dorsal habenular nucleus; PM: magoncellular preoptic nucleus; OC: optic chiasma; POA: preoptic area; TelV: telencephalic ventricle; VL: ventrolateral thalamic nuclei. Scale bar: 120 μm except in C and C’: 60 μm.

**Fig 4:**
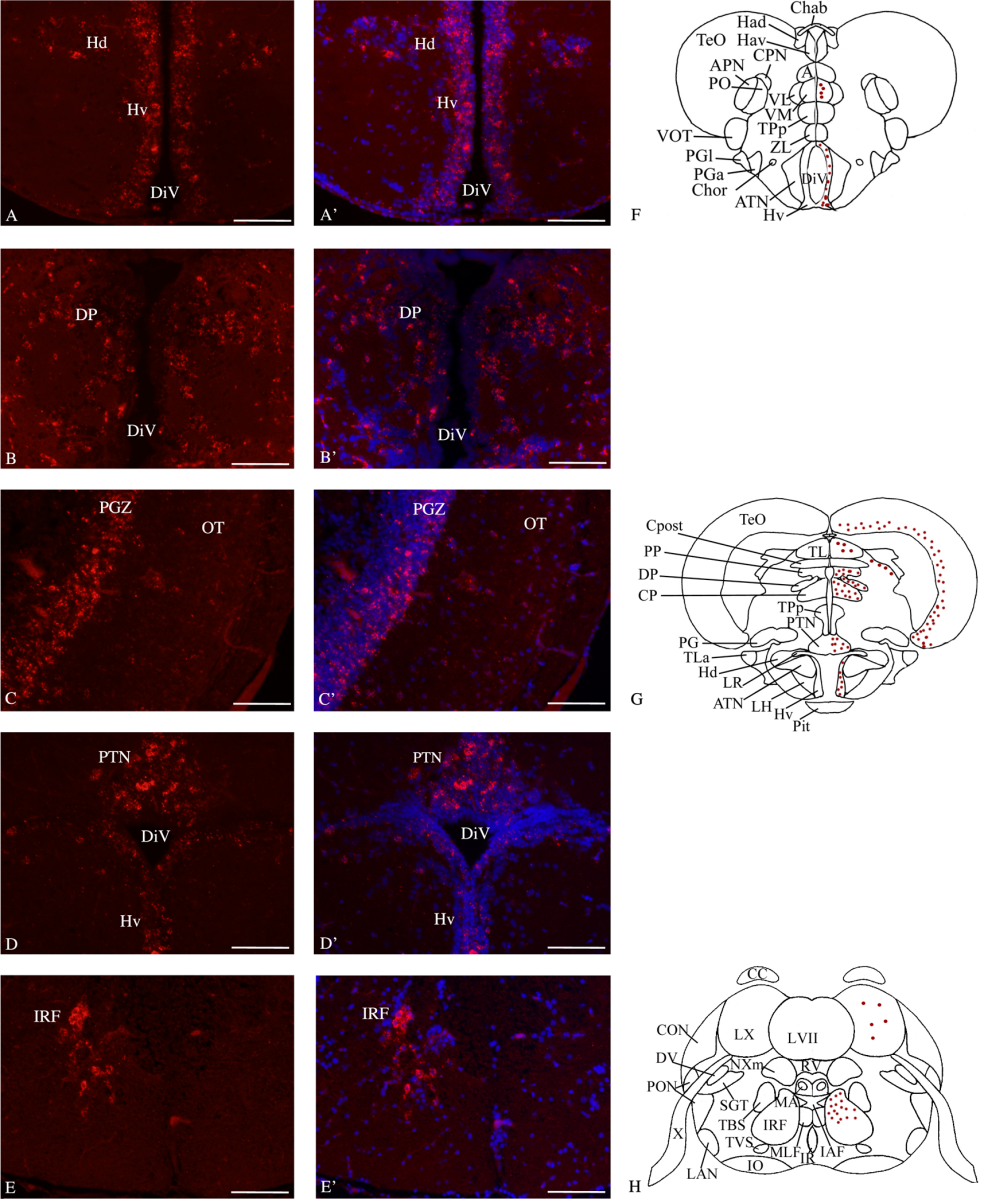
*Bdnf* mRNA distribution in cross-sections through the adult zebrafish mid- and hindbrain. In the ventral hypothalamus (**A, A’, F and G**), the dorsal thalamic region (**B, B’**), the optic tectum (**C, C’ and G**), the posterior tuberal nucleus (**D, D’ and G**) and medulla oblongata (**E, E’ and H**). In A', B', C', D' and E’, cell nuclei are labeled in blue with DAPI. F, G and H are representative sections taken from the zebrafish atlas (Wullimann et al., 1996). *Bdnf*-expressing cells are represented by red dots. DiV: diencephalic ventricle, DP: dorsal thalamic nucleus; Hd: dorsal zone of the periventricular hypothalamus; Hv: ventral zone of the periventricular hypothalamus; IRF: inferior reticular formation; OT: optic tectum; PGZ: periventricular gray zone of the optic tectum. PTN: posterior tuberal nucleus. Scale bar: 120 μm.

The distribution of *bdnf* mRNAs was studied on transverse brain sections of sexually mature males and females from the olfactory bulbs to the medulla oblongata. No labeling was observed with the sense probe (S2 Fig, [38]). The distribution of *bdnf* mRNAs, revealed by the in situ reaction with the antisense riboprobe, is shown in Table 2 and Figs 3 and 4.

**Table 2:** Expression of *bdnf* in the brain of adult zebrafish. + = few cells; ++ = moderate number of cells; +++ = numerous cells.

**Olfactory bulbs**	
Glomerular layer of olfactory bulb	++
External cellular layer of olfactory bulb	+
Internal cellular layer of olfactory bulb	+
Lateral olfactory tract	+
Medial olfactory tract	+
**Dorsal telencephalic area**	
Lateral zone of dorsal telencephalic area	++
Medial zone of dorsal telencephalic area	+++
Central zone of dorsal telencephalic area	+++
Dorsal zone of dorsal telencephalic area	++
Posterior zone of dorsal telencephalic area	+++
**Ventral telencephalic area**	
Entopeduncular nucleus, dorsal part	+
Postcommissural nucleus of ventral telencephalic area	+
**Preoptic area**	
Parvocellular preoptic nucleus, anterior part	++
Magnocellular preoptic nucleus	+
Parvocellular preoptic nucleus, posterior part	+
Subglomerular nucleus	+
**Epithalamus**	
Dorsal habenular nucleus	++
Ventral habenular nucleus	++
**Dorsal thalamus**	
Anterior thalamic nucleus	+
Posterior zone of dorsal telencephalic area	+
Central posterior thalamic nucleus	+
**Ventral thalamus**	
Ventromedial thalamic nucleus	+
Ventrolateral thalamic nucleus	++
**Posterior Tuberculum**	
Posterior tuberal nucleus	+++
Anterior preglomerular nucleus	++
Lateral preglomerular nucleus	++
Medial preglomerular nucleus	+
**Hypothalamus**	
Diffuse nucleus of the inferior lobe	+
Ventral zone of periventricular hypothalamus	++
Anterior tuberal nucleus	+
Lateral hypothalamic nucleus	++
Central nucleus of the inferior lobe	++
Dorsal zone of periventricular hypothalamus	+
Mammillary body	+
**Synencephalon**	
Nucleus of the medial longitudinal fascicle	+
Periventricular pretectal nucleus, ventral part	++
Periventricular pretectal nucleus, dorsal part	+++
**Optic Tectum**	
Periventricular grey zone of optic tectum	++
Deep white zone	+
Central zone of optic tectum	+
Superficial white zone	+
Longitudinal torus	+
**Torus semicircularis**	
Central nucleus of semicircular torus	+
**Tegmentum**	
Interpeduncular nucleus	+
Superior reticular formation	+
**Cerebellum**	
Granular cell layer	+
**Medulla oblongata**	
Secondary gustatory nucleus	++
Inferior reticular formation	++
Vagal lobe	+

The overall pattern of *bdnf* expression was identical in all animals. In particular, *bdnf* mRNAs were expressed in the same areas/regions in males and in females. There was no obvious difference between sexes, either in staining intensity and labeled-cells number. For this reason, micrographs presented in Figs 3 and 4 include both male and female brains. The olfactory bulbs displayed few *bdnf*-positive cells in the glomerular cell layer and in the external and internal cell layers (data not shown). A strong hybridization staining was observed in a large number of small round cells localized in the dorsal telencephalon particularly in its medial, lateral and posterior divisions (Fig 3A–3C’ and 3E). The ventral part of the telencephalon exhibited fewer and weakly labeled cells in the posterior zone and intensely stained cells in the entopedoncular nucleus (Fig 3A and 3B and 3E). In the diencephalon, we observed an intense positive signal in the parvocellular (Fig 3D and 3E), and magnocellular nuclei of the preoptic area and in the entopeduncular and suprachiasmatic nuclei (Fig 3G). *Bdnf* mRNAs are strongly expressed in cells of the habenula, specifically in its dorsal component (Fig 3H–3I) and in the ventrolateral and ventromedial nuclei of the ventral thalamus (Figs 3F and 3F’ and 4F), but the intensity of labeling was weaker than in the dorsal thalamic nuclei (see below). Transcripts were also abundantly reported in the anterior, dorsal, posterior and central posterior nuclei of the dorsal thalamus (Fig 4B and 4B’ and 4G). More ventrally, *bdnf* mRNAs were expressed in the posterior tuberal nucleus (Fig 4D and 4D’ and 4F) and preglomerular nuclei. In the hypothalamus, *bdnf* transcripts were abundantly expressed in periventricular nucleus of its ventral and dorsal part (Fig 4A and 4A’, 4D, 4D’, 4F and 4G), in the diffuse and central nuclei of the inferior lobe and in the mammilary body. Between diencephalon and mesencephalon, in the so-called synencephalon, the nucleus of the medial longitudinal fascicle displayed few *bdnf*-positive cells, whereas *bdnf* mRNAs were highly expressed in the dorsal and ventral periventricular pretectal nuclei. In the mesencephalon, *bdnf* mRNAs were also observed in the torus longitudinalis. The periventricular gray zone of the optic tectum exhibited numerous cells expressing *bdnf* trancripts (Fig 4C and 4C’ and 4G). In the tegmentum, *bdnf* mRNA were observed in the central nucleus of the torus semicircularis and in the interpeduncular nucleus. In the medulla oblongata, *bdnf* mRNAs were highly expressed in the secondary gustatory nucleus and in few cells of the vagal lobe. Finally, large *bdnf*-positive perikarya were seen in the superior and inferior reticular formation (Fig 4E and 4E’ and 4H).

### *Bdnf*-expressing cells do not proliferate and are not radial glial cells but neurons

**Fig 5:**
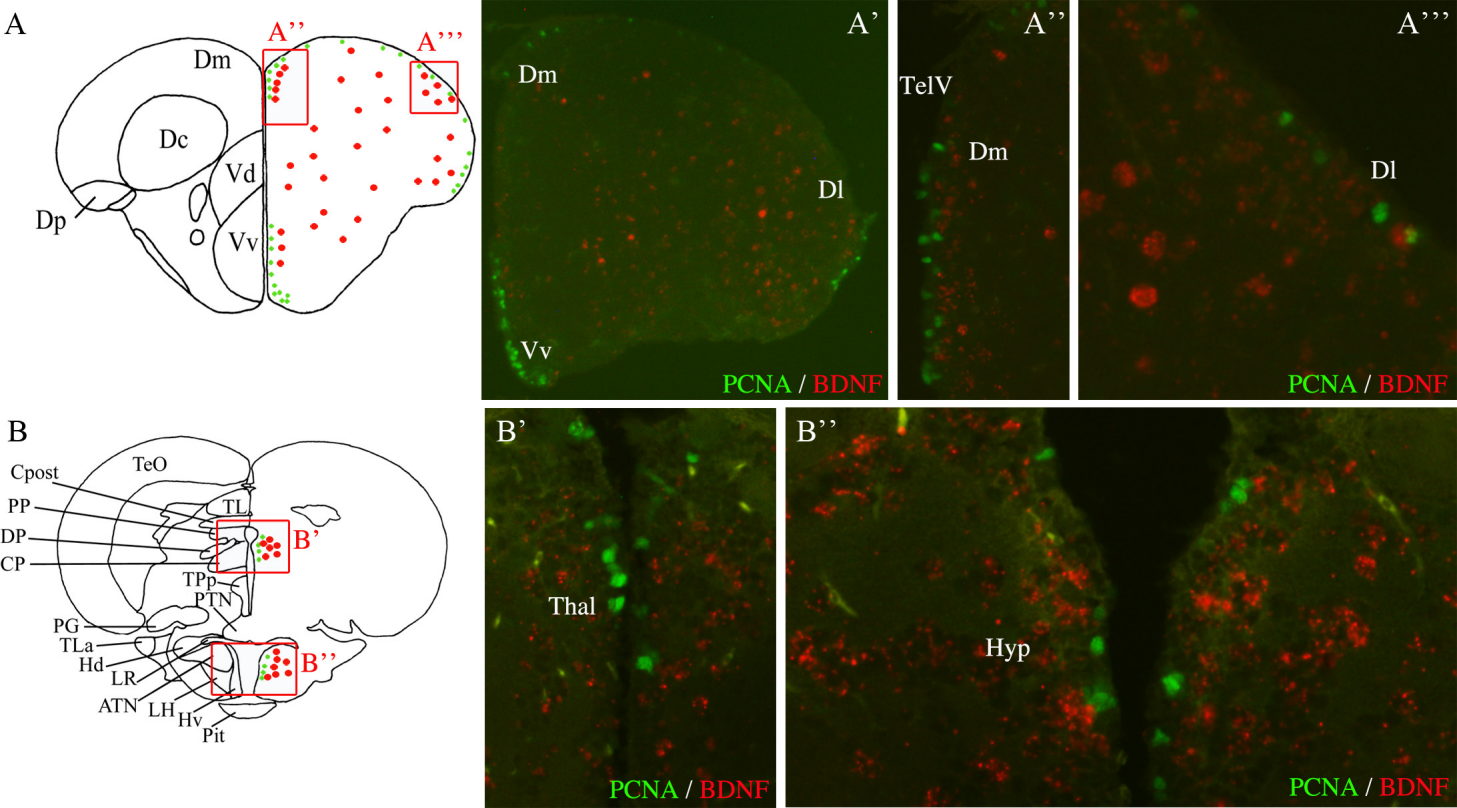
Immunohistochemical characterization of *bdnf*-expressing cells in adult zebrafish brain. Double staining for *bdnf* mRNA (red) and PCNA protein (green) on cross-sections through the telencephalon (**A to A”‘**), the thalamus (**B and B’**) and the ventral hypothalamus (**B and B”**). A and B are representative sections taken from the zebrafish atlas (Wullimann et al., 1996). *Bdnf*-expressing cells are represented by red dots and PCNA-labeled cells are green dots. Dl: lateral zone of the dorsal telencephalon; Dm: medial zone of the dorsal telencephalon; Hyp: hypothalamus; TelV: telencephalic ventricle; Thal: thalamus; Vv: ventral zone of the ventral telencephalon. Scale bar = 200 μm in A’; 100 μm in A”; 50 μm in A”‘.

In order to identify the nature of *bdnf*-expressing cells in the brain of adult zebrafish, we performed *bdnf* in situ hybridization in combination with immunohistochemistry using antibodies directed against different types of markers (proliferation, neuronal or glial markers), as described above. *Bdnf*-expressing cells were visualized close to the ventricular surface in many regions such as the telencephalon, the preoptic area, the hypothalamus, the thalamus, and the optic tectum. Previous studies showed that in the brain of adult zebrafish, many proliferating cells were located along the ventricles in those regions [61, 63]. To investigate if cells undergoing proliferation could be *bdnf*-expressing cells, we performed PCNA labeling in combination with *bdnf* in situ hybridization. As expected, PCNA-labeled cells were positioned along the ventricular cavities in the telencephalon (Fig 5A–5A”‘), in the thalamus (Fig 5B and 5B’) and in the hypothalamus (Fig 5B and 5B”). In those regions, *bdnf*-expressing cells were observed very close to PCNA-labeled nuclei, but *bdnf* mRNAs were never expressed by the proliferating cells (PCNA-positive cells).

**Fig 6:**
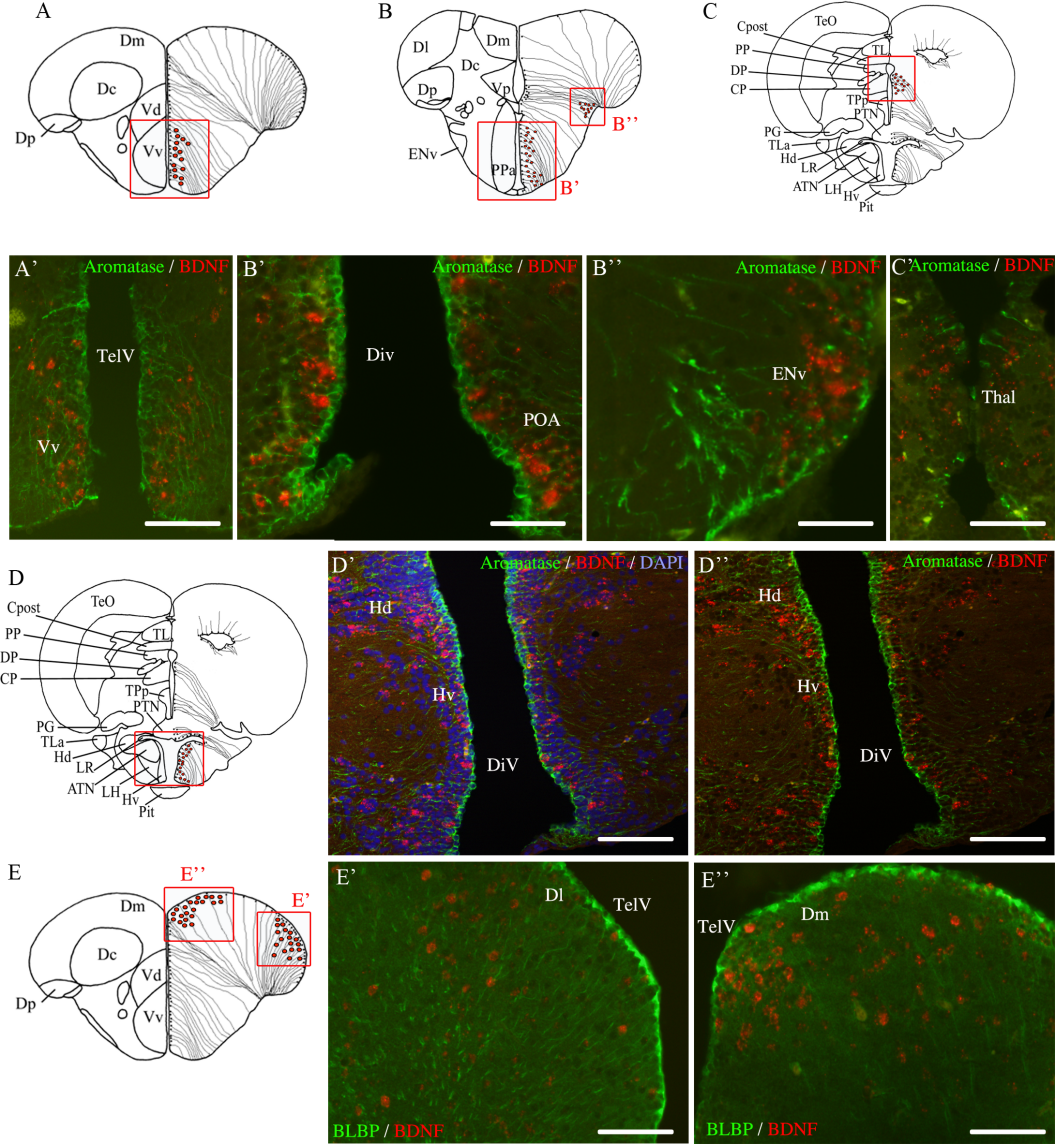
Immunohistochemical characterization of *bdnf*-expressing cells in adult zebrafish brain. A, B, C, D and E are representative sections taken from the zebrafish atlas (Wullimann et al., 1996). *Bdnf*-expressing cells are represented by red dots and Aromatase B (**A-D**) or BLBP-labeled cells (**E**) are represented by black dots with thin lines indicating radial glia cytoplasmic processes. Double staining for *bdnf* mRNA (red) and Aromatase B protein (green) on cross-sections through the telencephalon (**A-A’**), the preoptic area (**B-B’**), the entopedoncular nucleus (**B-B”**), the thalamus (**C-C’**) and the ventral hypothalamus (**D-D”**). Double staining for *bdnf* mRNA (red) and BLBP protein (green) on cross-sections through the telencephalon (**E-E”**). DiV: diencephalic ventricle; Dl: lateral zone of the dorsal telencephalon; Dm: medial zone of the dorsal telencephalon; ENv: endopedoncular nucleus; Hd: dorsal zone of the periventricular hypothalamus; Hv: ventral zone of the periventricular hypothalamus; POA: preoptic area; TelV: telencephalic ventricle; Vv: ventral zone of the ventral telencephalon. D’ and D” are obtained with an Apotome-equipped Zeiss. Scale bar = 60 μm in A’, C’, D’, D”, E’ and E”; 30 μm in B’ and B”.

**Fig 7:**
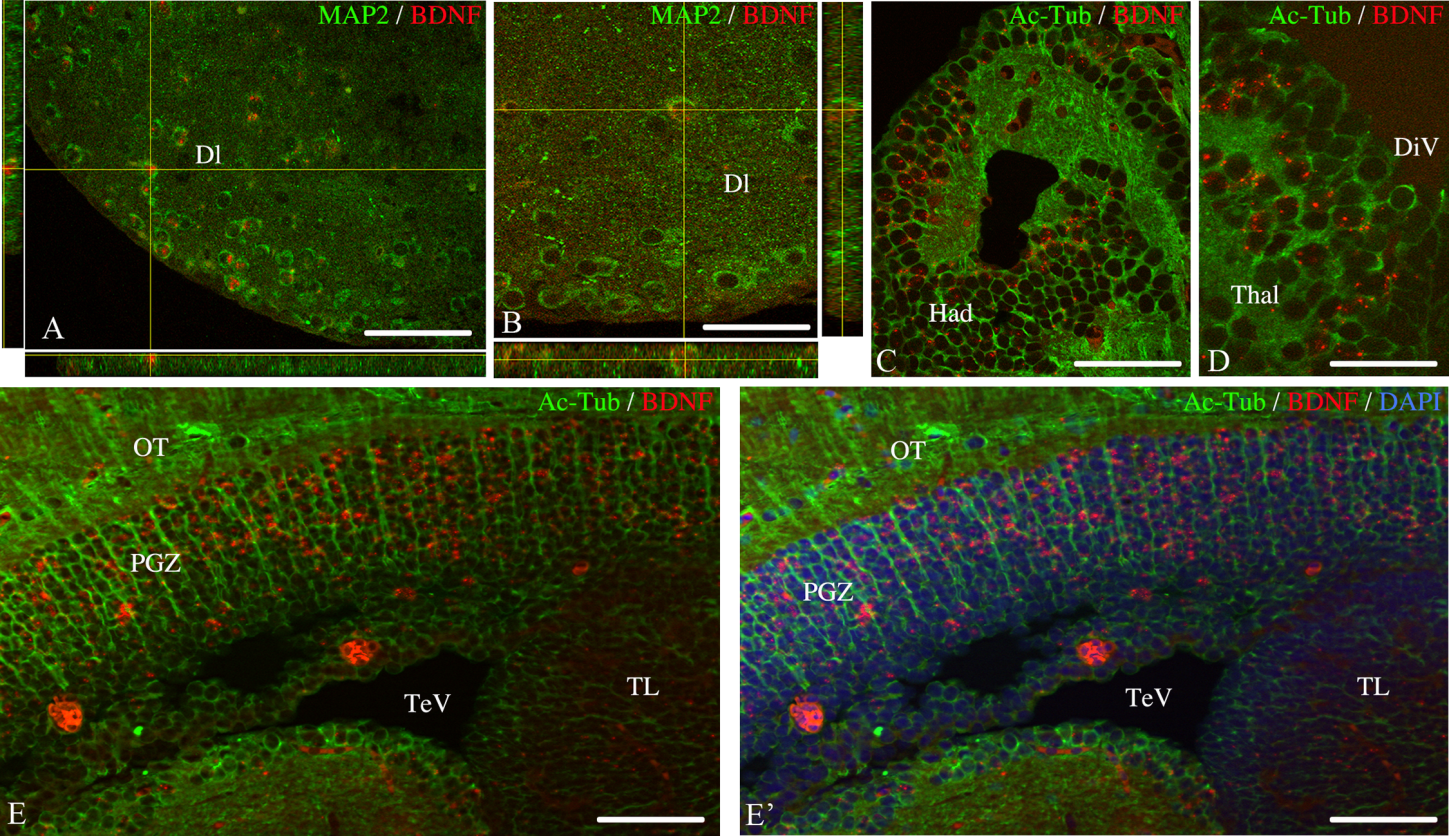
Transverse sections of adult zebrafish brain showing co-expression of *bdnf* mRNA with neuronal markers. Orthogonal projections of Z-stacks from telencephalon (11 sections of 0.5 μm) evidencing the expression of *bdnf* (red) in neurons identified by MAP2 protein (green) (**A and B**). Double staining for *bdnf* mRNA (red) and acetylated-tubuline (green) in the habenula (**C**), the thalamus (**D**) and the optic tectum (**E-E’**). In E’, cell nulei are labeled in blue with DAPI. Dl: lateral zone of the dorsal telencephalon; Had: dorsal habenular nucleus; OT: optic tectum; PGZ: periventricular gray zone of the optic tectum; TelV: telencephalic ventricle; Thal: thalamus; TL: torus longitudinalis. A, B, C and D were obtained with the confocal microscope. E and E’ were obtained with the Apotome. Scale bar: 50 μm in E and E’; 40 μm in A and C; 25 μm in B and D.

To test whether some of the *bdnf*-expressing cells could be radial glial cells, we combined *bdnf* hybridization with immunohistochemistry against aromatase B or BLBP, two well-established markers of radial glial cells in fish and other vertebrates [61, 76–78]. As shown in Fig 6, aromatase B-positive cells never co-expressed *bdnf* mRNAs in the ventral telencephalon (Fig 6A and 6A’), preoptic area (Fig 6B and 6B’), entopeduncular nucleus (Fig 6B and 6B”) and thalamus (Fig 6C and 6C’). Analysis of sections from the entire brain using an Apotome-equipped Zeiss microscope did not provide any evidence for expression of *bdnf* mRNAs in radial glia (Fig 6D–6D”). Similarly, double staining with BLBP antibodies failed to demonstrate any *bdnf* mRNAs in radial glial cells (Fig 6E–6E”). In contrast, antibodies against neuronal markers (MAP2 and acetylated tubulin) indicated that *bdnf* mRNAs were expressed in cells with neuronal phenotype in all brain regions investigated. Immunohistochemistry using anti-MAP2 antibody revealed that *bdnf* mRNAs was exclusively present in neuronal cells as shown in the telencephalon with confocal orthogonal projections (Fig 7A and 7B). Immunohistochemistry using anti-acetylated-tubulin antibody confirmed that *bdnf* was expressed only in neuronal cells in the telencephalon, habenula (Fig 7C), thalamus (Fig 7D), hypothalamus and optic tectum (Fig 7E and 7E’).

## Discussion

In the present study, we reported, for the first time, that *bdnf* mRNAs are consistently expressed in the brain of larval and adult zebrafish. Our results also demonstrated that cells expressing *bdnf* mRNAs do not correspond to radial glial cells or progenitor cells but rather to neurons.

The present study shows that *bdnf* mRNAs are widely expressed in the brain of 7 days old larvae, which is in agreement with the data previously obtained by RT-PCR and showing that *bdnf* expression is maternally inherited, drops down at 1 day post-fertilization (dpf) and then progressively increases from 1 to 8 dpf [38]. Our results bring new information by showing that the strongest signal is observed in the dorsal telencephalon (pallium), the preoptic region, the thalamus and the optic tectum. In general, our expression is in good agreement with what has been shown by previous authors [38]. Indeed, the distribution of *bdnf* mRNAs was addressed by in toto hybridization and established that *bdnf* was strongly expressed in the pallium, hypothalamus, posterior tuberculum and optic tectum at 1, 2 and 3 dpf. However, there were no detailed information on the precise structures expressing these messengers. In addition, at 7 dpf, *bdnf* mRNAs level seems to be lower, possibly due to a problem of access of the probe to the brain [38]. Our results show that in all cases, cells expressing *bdnf* mRNAs were located in the parenchyma and are identified as neurons. Furthermore, *bdnf*-expressing cells never exhibited PCNA, aromatase B or BLBP staining, indicating that *bdnf* mRNAs are not expressed in radial glia and/or progenitors. Although the function of BDNF in the developing brain of zebrafish was not addressed in the present study, these data suggest that this factor could be involved in the differentiation and maintenance of newborn neurons, mainly in the forebrain.

In adult zebrafish, *bdnf* expression has a similar distribution as in the larvae with the most prominent staining in dorsal telencephalon, preoptic area, dorsal thalamus, posterior tuberculum, hypothalamus, synencephalon and optic tectum. A more diffuse and weaker labelling is detected in other brain regions. The pattern of distribution of *bdnf* mRNAs is relatively similar to that reported in the European eel and the Turquoise killifish [35, 37] with some slight differences regarding the ventral telencephalon, diencephalon, tegmentum and rhombencephalon. Compared to the European eel and the turquoise killifish, *bdnf* expression in zebrafish is less expressed in the ventral telencephalon. By contrast, the staining in the diencephalon appears more widespread in the zebrafish than in the eel. Some slight differences were seen in other diencephalic nuclei, such as nucleus corticalis and nucleus glomerulosus, which were intensely positive in the killifish and are lacking in the brain of zebrafish. Finally, the tegmentum and rhombencephalon of zebrafish showed fewer *bdnf*-expressing cells than eel and turquoise killifish. Thus, we can conclude that, in zebrafish, *bdnf* is expressed mostly in the forebrain and just in few areas of midbrain and hindbrain.

The localization of *bdnf* transcripts in adult zebrafish is similar in both sexes, despite the fact that sex hormones have been recognized to influence the expression of *bdnf* through a combination of genomic and epigenetic mechanisms [79, 80]. The general idea about the regulation of BDNF (protein and gene activation) by estradiol is that estradiol increases *bdnf* mRNAs levels. However, if we examine at the literature into more detail, the impacts of estrogens on *bdnf* mRNAs are complex and fluctuate according to the species, age, brain region and treatment [79–81]. Preliminary experiments performed in the laboratory and based on RT-PCR in whole brains failed to show any significant effect of estradiol exposure (10^-7^M to 10^-10^M) on *bdnf* expression in 7dpf zebrafish larvae.

Because in some regions, *bdnf*-expressing cells in adults were located close to the ventricular surface, we examined whether such cells could correspond to radial glia cells. Indeed, we and others have shown that in adult fish radial glial cells line entirely the ventricles [61, 70, 82]. Furthermore, it is now recognized that radial glial cells are progenitors and sustain the constant growth of the brain throughout life [61, 83]. To investigate if radial glial cells express or not *bdnf* transcripts, we used aromatase B and BLBP immunohistochemistry in combination with *bdnf* in situ hybridization [70, 71, 84]. Interestingly, we could not detect *bdnf* mRNAs/aromatase B or *bdnf* mRNAs/BLBP co-staining, suggesting that radial glial cells do not express *bdnf* under physiological conditions. However, in mammals, *bdnf* expression has been documented in glial cells under pathological conditions, in particular after brain lesions or around amyloid plaques [28, 85, 86]. We previously developed a model of telencephalic lesion in adult zebrafish and showed that proliferation increased rapidly after injury in the parenchyma of the injured telencephalon [68]. Thus, it would be interesting to investigate, in this model, if *bdnf* expression could be induced in glial cells after brain injuries (as in mammals). Double staining with the cell proliferation marker PCNA failed to show any co-expression of *bdnf* mRNAs and PCNA in the same cells. However, this does not rule out the possibility that they may express *bdnf* mRNAs later. A study performed in zebra finch showed that, 15 days after bromodeoxyuridine injection (BrdU, a marker for new born cells), BDNF protein is present in new generated cells labeled with BrdU [87]. In our study, double staining with neuronal makers, notably HuC/D, MAP2 and acetylated-tubulin clearly showed that *bdnf* -expressing cells have a neuronal phenotype in the brain of adult zebrafish. This finding is consistent with the fact that BDNF is a factor of neuronal origin in mammals [22, 24, 29]. The neuronal marker HuC/D specifically characterizes early-differentiated neurons [88], whereas the markers MAP2 and acetylated-tubulin characterize both early and late differentiated neurons [89, 90]. Therefore, we can assume that *bdnf* mRNAs could be expressed in the early stages of differentiation processes.

It is interesting to note that the distribution of *bdnf* mRNAs in the brain of zebrafish is quite similar to that reported in mammals, notably in rat, mouse and pig [21–26]. Indeed, strong *bdnf* expression was reported in the cortex and hippocampus, two pallial structures. Because the telencephalon of fish develops by eversion, it is usually considered that the hippocampus equivalent in zebrafish is the dorsolateral region of the dorsal telencephalon [91–93], in which abundant *bdnf* mRNAs are detected. It is also noteworthy that the central part of the telencephalon, regarded as the presumptive equivalent of the isocortex of mammals, also strongly expresses *bdnf* [22]. Similarly, the preoptic area and in particular the magnocellular neurons express *bdnf* trancripts, similar to their mammalian counterparts, the paraventricular and supraoptic nuclei. Other structures exhibiting *bdnf* mRNAs in both fishes and mammals include the habenula, the thalamic region, the mediobasal hypothalamus and the optic tectum (inferior colliculus). These similarities suggest that BDNF functions are conserved between fishes and mammals and have emerged early in the vertebrate lineage. Studies in Drosophila have recently reported the existence of a neurotrophin-related gene (*DNT1*) [94]. According to this study, this gene would represent the ancestor of the neurotrophin family that diversified through whole genome duplications. Interestingly, in Drosophila, DNT1 exerts trophic and maintenance functions on neurons.

In conclusion, this study presents a detailed pattern of *bdnf* mRNAs distribution in the brain of larva and adult zebrafish under normal conditions. We show that *bdnf* transcripts are most abundant in forebrain regions (that are homolog to *bdnf*-expressing areas in the mammalian brain). We also bring evidences that *bdnf* mRNAs are expressed in neurons and not in radial glial cells or progenitor cells. As BDNF contributes to the organization and plasticity of neural network in the intact brain in mammals, further studies in fish should aim at investigating its potential roles in the developing and adult brain in physiological conditions, but also in the case of brain recovery after injuries.

## Supporting Information

S1 Fig The *bdnf* sense riboprobe did not generate any signal in the brain of 7 days old zebrafish. Telencephalon (**A**), Preoptic area (**B**), Optic Tectum (**B and C**). Hyp: hypothalamus; POA: preoptic area; OR: olfactory rosettes; OT: optic tectum. Teg: tegmentum; Tel: telencephalon. Scale bar: 120 μm in A and C. Scale bar: 60 μm in B.
(TIF)

S2 Fig The *bdnf* sense riboprobe did not generate any signal in the brain of adult zebrafish. Telencephalon (**A and B**), Preoptic area (**C**), Thalamus (**D**), Hypothalamus (**E**) and Optic Tectum (F). Dc: central zone of the dorsal telencephalon; DiV: diencephalic ventricle; Dl: lateral zone of the dorsal telencephalon; Dm: medial zone of the dorsal telencephalon; Dp: posterior zone of the dorsal telencephalon; DP: dorsal thalamic nucleus; Hd: dorsal zone of the periventricular hypothalamus; Hv: ventral zone of the periventricular hypothalamus; OC: optic chiasma; OT: optic tectum; PGZ: periventricular gray zone of the optic tectum; POA: preoptic area. Scale bar: 120 μm in A, C. Scale bar: 60 μm in B, D, E, F.
(TIF)

## References

[1] Chao MV. Neurotrophins and their receptors: a convergence point for many signalling pathways. Nature reviews Neuroscience. 2003;4(4):299–309. Epub 2003/04/03. 10.1038/nrn1078 . 12671646

[2] Huang EJ, Reichardt LF. Neurotrophins: roles in neuronal development and function. Annual review of neuroscience. 2001;24:677–736. Epub 2001/08/25. 10.1146/annurev.neuro.24.1.677 11520916 PMC2758233

[3] Carter AR, Berry EM, Segal RA. Regional expression of p75NTR contributes to neurotrophin regulation of cerebellar patterning. Molecular and cellular neurosciences. 2003;22(1):1–13. Epub 2003/02/22. . 12595234 10.1016/s1044-7431(02)00015-5

[4] Cosgaya JM, Chan JR, Shooter EM. The neurotrophin receptor p75NTR as a positive modulator of myelination. Science (New York, NY). 2002;298(5596):1245–8. Epub 2002/11/09. 10.1126/science.1076595 . 12424382

[5] Bamji SX, Majdan M, Pozniak CD, Belliveau DJ, Aloyz R, Kohn J, et al The p75 neurotrophin receptor mediates neuronal apoptosis and is essential for naturally occurring sympathetic neuron death. The Journal of cell biology. 1998;140(4):911–23. Epub 1998/03/21. 9472042 10.1083/jcb.140.4.911 PMC2141754

[6] Boyd JG, Gordon T. A dose-dependent facilitation and inhibition of peripheral nerve regeneration by brain-derived neurotrophic factor. The European journal of neuroscience. 2002;15(4):613–26. Epub 2002/03/12. . 11886442 10.1046/j.1460-9568.2002.01891.x

[7] Yamada K, Mizuno M, Nabeshima T. Role for brain-derived neurotrophic factor in learning and memory. Life sciences. 2002;70(7):735–44. Epub 2002/02/09. . 11833737 10.1016/s0024-3205(01)01461-8

[8] Sambataro F, Murty VP, Lemaitre HS, Reed JD, Das S, Goldberg TE, et al BDNF modulates normal human hippocampal ageing [corrected]. Molecular psychiatry. 2010;15(2):116–8. Epub 2010/01/26. 10.1038/mp.2009.64 20098437 PMC3073456

[9] von Bohlen und Halbach O. Involvement of BDNF in age-dependent alterations in the hippocampus. Frontiers in aging neuroscience. 2010;2 Epub 2010/10/14. 10.3389/fnagi.2010.00036 20941325 PMC2952461

[10] Zhang F, Kang Z, Li W, Xiao Z, Zhou X. Roles of brain-derived neurotrophic factor/tropomyosin-related kinase B (BDNF/TrkB) signalling in Alzheimer's disease. Journal of clinical neuroscience: official journal of the Neurosurgical Society of Australasia. 2012;19(7):946–9. Epub 2012/05/23. 10.1016/j.jocn.2011.12.022 . 22613489

[11] Fumagalli F, Racagni G, Riva MA. The expanding role of BDNF: a therapeutic target for Alzheimer's disease? The pharmacogenomics journal. 2006;6(1):8–15. Epub 2005/11/30. 10.1038/sj.tpj.6500337 . 16314887

[12] Peng S, Garzon DJ, Marchese M, Klein W, Ginsberg SD, Francis BM, et al Decreased brain-derived neurotrophic factor depends on amyloid aggregation state in transgenic mouse models of Alzheimer's disease. The Journal of neuroscience: the official journal of the Society for Neuroscience. 2009;29(29):9321–9. Epub 2009/07/25. 10.1523/jneurosci.4736-08.2009 19625522 PMC3411546

[13] Cohen-Cory S, Kidane AH, Shirkey NJ, Marshak S. Brain-derived neurotrophic factor and the development of structural neuronal connectivity. Developmental neurobiology. 2010;70(5):271–88. Epub 2010/02/27. 10.1002/dneu.20774 20186709 PMC2893579

[14] Aid T, Kazantseva A, Piirsoo M, Palm K, Timmusk T. Mouse and rat BDNF gene structure and expression revisited. Journal of neuroscience research. 2007;85(3):525–35. Epub 2006/12/07. 10.1002/jnr.21139 17149751 PMC1878509

[15] Tao X, West AE, Chen WG, Corfas G, Greenberg ME. A calcium-responsive transcription factor, CaRF, that regulates neuronal activity-dependent expression of BDNF. Neuron. 2002;33(3):383–95. Epub 2002/02/08. . 11832226 10.1016/s0896-6273(01)00561-x

[16] Rattiner LM, Davis M, French CT, Ressler KJ. Brain-derived neurotrophic factor and tyrosine kinase receptor B involvement in amygdala-dependent fear conditioning. The Journal of neuroscience: the official journal of the Society for Neuroscience. 2004;24(20):4796–806. Epub 2004/05/21. 10.1523/jneurosci.5654-03.2004 . 15152040 PMC6729469

[17] Kidane AH, Heinrich G, Dirks RP, de Ruyck BA, Lubsen NH, Roubos EW, et al Differential neuroendocrine expression of multiple brain-derived neurotrophic factor transcripts. Endocrinology. 2009;150(3):1361–8. Epub 2008/11/15. 10.1210/en.2008-0993 . 19008311

[18] Heinrich G, Pagtakhan CJ. Both 5' and 3' flanks regulate Zebrafish brain-derived neurotrophic factor gene expression. BMC neuroscience. 2004;5:19 Epub 2004/05/22. 10.1186/1471-2202-5-19 15153250 PMC442124

[19] Tettamanti G, Cattaneo AG, Gornati R, de Eguileor M, Bernardini G, Binelli G. Phylogenesis of brain-derived neurotrophic factor (BDNF) in vertebrates. Gene. 2010;450(1–2):85–93. Epub 2009/11/03. 10.1016/j.gene.2009.07.023 . 19879341

[20] Maisonpierre PC, Belluscio L, Friedman B, Alderson RF, Wiegand SJ, Furth ME, et al NT-3, BDNF, and NGF in the developing rat nervous system: parallel as well as reciprocal patterns of expression. Neuron. 1990;5(4):501–9. Epub 1990/10/01. . 1688327 10.1016/0896-6273(90)90089-x

[21] Hofer M, Pagliusi SR, Hohn A, Leibrock J, Barde YA. Regional distribution of brain-derived neurotrophic factor mRNA in the adult mouse brain. The EMBO journal. 1990;9(8):2459–64. Epub 1990/08/01. 2369898 10.1002/j.1460-2075.1990.tb07423.x PMC552273

[22] Castren E, Thoenen H, Lindholm D. Brain-derived neurotrophic factor messenger RNA is expressed in the septum, hypothalamus and in adrenergic brain stem nuclei of adult rat brain and is increased by osmotic stimulation in the paraventricular nucleus. Neuroscience. 1995;64(1):71–80. Epub 1995/01/01. . 7708216 10.1016/0306-4522(94)00386-j

[23] Yan Q, Rosenfeld RD, Matheson CR, Hawkins N, Lopez OT, Bennett L, et al Expression of brain-derived neurotrophic factor protein in the adult rat central nervous system. Neuroscience. 1997;78(2):431–48. Epub 1997/05/01. . 9145800 10.1016/s0306-4522(96)00613-6

[24] Schmidt-Kastner R, Wetmore C, Olson L. Comparative study of brain-derived neurotrophic factor messenger RNA and protein at the cellular level suggests multiple roles in hippocampus, striatum and cortex. Neuroscience. 1996;74(1):161–83. Epub 1996/09/01. . 8843085 10.1016/0306-4522(96)00093-0

[25] Conner JM, Lauterborn JC, Yan Q, Gall CM, Varon S. Distribution of brain-derived neurotrophic factor (BDNF) protein and mRNA in the normal adult rat CNS: evidence for anterograde axonal transport. The Journal of neuroscience: the official journal of the Society for Neuroscience. 1997;17(7):2295–313. Epub 1997/04/01. . 9065491 10.1523/JNEUROSCI.17-07-02295.1997 PMC6573520

[26] Wetmore C, Ernfors P, Persson H, Olson L. Localization of brain-derived neurotrophic factor mRNA to neurons in the brain by in situ hybridization. Experimental neurology. 1990;109(2):141–52. Epub 1990/08/01. . 2379553 10.1016/0014-4886(90)90068-4

[27] Katoh-Semba R, Takeuchi IK, Semba R, Kato K. Distribution of brain-derived neurotrophic factor in rats and its changes with development in the brain. Journal of neurochemistry. 1997;69(1):34–42. Epub 1997/07/01. . 9202291 10.1046/j.1471-4159.1997.69010034.x

[28] Murer MG, Boissiere F, Yan Q, Hunot S, Villares J, Faucheux B, et al An immunohistochemical study of the distribution of brain-derived neurotrophic factor in the adult human brain, with particular reference to Alzheimer's disease. Neuroscience. 1999;88(4):1015–32. Epub 1999/05/21. . 10336117 10.1016/s0306-4522(98)00219-x

[29] Quartu M, Serra MP, Boi M, Melis T, Ambu R, Del Fiacco M. Brain-derived neurotrophic factor (BDNF) and polysialylated-neural cell adhesion molecule (PSA-NCAM): codistribution in the human brainstem precerebellar nuclei from prenatal to adult age. Brain research. 2010;1363:49–62. Epub 2010/10/12. 10.1016/j.brainres.2010.09.106 . 20932956

[30] Tang YP, Wade J. 17beta-estradiol regulates the sexually dimorphic expression of BDNF and TrkB proteins in the song system of juvenile zebra finches. PloS one. 2012;7(8):e43687 Epub 2012/09/07. 10.1371/journal.pone.0043687 22952738 PMC3432032

[31] Tang YP, Wade J. Developmental changes in BDNF protein in the song control nuclei of zebra finches. Neuroscience. 2013;250:578–87. Epub 2013/08/08. 10.1016/j.neuroscience.2013.07.062 23920158 PMC3789383

[32] Brenowitz EA. Testosterone and brain-derived neurotrophic factor interactions in the avian song control system. Neuroscience. 2013;239:115–23. Epub 2012/11/06. 10.1016/j.neuroscience.2012.09.023 23123886 PMC3612365

[33] Duprey-Diaz MV, Soto I, Blagburn JM, Blanco RE. Changes in brain-derived neurotrophic factor and trkB receptor in the adult Rana pipiens retina and optic tectum after optic nerve injury. The Journal of comparative neurology. 2002;454(4):456–69. Epub 2002/11/28. 10.1002/cne.10451 . 12455009

[34] Wang L, Calle M, Roubos EW. Brain-derived neurotrophic factor in the hypothalamo-hypophyseal system of Xenopus laevis. Annals of the New York Academy of Sciences. 2005;1040:512–4. Epub 2005/05/14. 10.1196/annals.1327.106 . 15891104

[35] Dalton VS, Borich SM, Murphy P, Roberts BL. Brain-derived neurotrophic factor mRNA expression in the brain of the teleost fish, Anguilla anguilla, the European Eel. Brain, behavior and evolution. 2009;73(1):43–58. Epub 2009/02/28. 10.1159/000204962 . 19246895

[36] Vissio PG, Canepa MM, Maggese MC. Brain-derived neurotrophic factor (BDNF)-like immunoreactivity localization in the retina and brain of Cichlasoma dimerus (Teleostei, Perciformes). Tissue & cell. 2008;40(4):261–70. Epub 2008/03/18. 10.1016/j.tice.2008.01.001 . 18343472

[37] D'Angelo L, De Girolamo P, Lucini C, Terzibasi ET, Baumgart M, Castaldo L, et al Brain-derived neurotrophic factor: mRNA expression and protein distribution in the brain of the teleost Nothobranchius furzeri. The Journal of comparative neurology. 2014;522(5):1004–30. Epub 2013/08/29. 10.1002/cne.23457 . 23983038

[38] De Felice E, Porreca I, Alleva E, De Girolamo P, Ambrosino C, Ciriaco E, et al Localization of BDNF expression in the developing brain of zebrafish. Journal of anatomy. 2014;224(5):564–74. 10.1111/joa.12168 . 24588510 PMC3981499

[39] Ip NY, Li Y, Yancopoulos GD, Lindsay RM. Cultured hippocampal neurons show responses to BDNF, NT-3, and NT-4, but not NGF. The Journal of neuroscience: the official journal of the Society for Neuroscience. 1993;13(8):3394–405. Epub 1993/08/01. . 7688038 10.1523/JNEUROSCI.13-08-03394.1993 PMC6576536

[40] Lindholm D, Carroll P, Tzimagiogis G, Thoenen H. Autocrine-paracrine regulation of hippocampal neuron survival by IGF-1 and the neurotrophins BDNF, NT-3 and NT-4. The European journal of neuroscience. 1996;8(7):1452–60. Epub 1996/07/01. . 8758952 10.1111/j.1460-9568.1996.tb01607.x

[41] Zigova T, Pencea V, Wiegand SJ, Luskin MB. Intraventricular administration of BDNF increases the number of newly generated neurons in the adult olfactory bulb. Molecular and cellular neurosciences. 1998;11(4):234–45. Epub 1998/07/24. 10.1006/mcne.1998.0684 . 9675054

[42] Pencea V, Bingaman KD, Wiegand SJ, Luskin MB. Infusion of brain-derived neurotrophic factor into the lateral ventricle of the adult rat leads to new neurons in the parenchyma of the striatum, septum, thalamus, and hypothalamus. The Journal of neuroscience: the official journal of the Society for Neuroscience. 2001;21(17):6706–17. Epub 2001/08/23. . 11517260 10.1523/JNEUROSCI.21-17-06706.2001 PMC6763082

[43] Scharfman H, Goodman J, Macleod A, Phani S, Antonelli C, Croll S. Increased neurogenesis and the ectopic granule cells after intrahippocampal BDNF infusion in adult rats. Experimental neurology. 2005;192(2):348–56. Epub 2005/03/10. 10.1016/j.expneurol.2004.11.016 . 15755552

[44] Galvao RP, Garcia-Verdugo JM, Alvarez-Buylla A. Brain-derived neurotrophic factor signaling does not stimulate subventricular zone neurogenesis in adult mice and rats. The Journal of neuroscience: the official journal of the Society for Neuroscience. 2008;28(50):13368–83. Epub 2008/12/17. 10.1523/jneurosci.2918-08.2008 19074010 PMC2659623

[45] Ernfors P, Lee KF, Jaenisch R. Mice lacking brain-derived neurotrophic factor develop with sensory deficits. Nature. 1994;368(6467):147–50. Epub 1994/03/10. 10.1038/368147a0 . 8139657

[46] Conover JC, Erickson JT, Katz DM, Bianchi LM, Poueymirou WT, McClain J, et al Neuronal deficits, not involving motor neurons, in mice lacking BDNF and/or NT4. Nature. 1995;375(6528):235–8. Epub 1995/05/18. 10.1038/375235a0 . 7746324

[47] Lee J, Duan W, Mattson MP. Evidence that brain-derived neurotrophic factor is required for basal neurogenesis and mediates, in part, the enhancement of neurogenesis by dietary restriction in the hippocampus of adult mice. Journal of neurochemistry. 2002;82(6):1367–75. Epub 2002/10/02. . 12354284 10.1046/j.1471-4159.2002.01085.x

[48] Chan JP, Cordeira J, Calderon GA, Iyer LK, Rios M. Depletion of central BDNF in mice impedes terminal differentiation of new granule neurons in the adult hippocampus. Molecular and cellular neurosciences. 2008;39(3):372–83. Epub 2008/08/23. 10.1016/j.mcn.2008.07.017 18718867 PMC2652348

[49] Hicks RR, Numan S, Dhillon HS, Prasad MR, Seroogy KB. Alterations in BDNF and NT-3 mRNAs in rat hippocampus after experimental brain trauma. Brain research Molecular brain research. 1997;48(2):401–6. Epub 1997/10/23. . 9332737 10.1016/s0169-328x(97)00158-7

[50] Wang Y, Hameed MQ, Rakhade SN, Iglesias AH, Muller PA, Mou DL, et al Hippocampal immediate early gene transcription in the rat fluid percussion traumatic brain injury model. Neuroreport. 2014;25(12):954–9. Epub 2014/07/01. 10.1097/wnr.0000000000000219 . 24978397

[51] Rostami E, Krueger F, Plantman S, Davidsson J, Agoston D, Grafman J, et al Alteration in BDNF and its receptors, full-length and truncated TrkB and p75(NTR) following penetrating traumatic brain injury. Brain research. 2014;1542:195–205. Epub 2013/11/07. 10.1016/j.brainres.2013.10.047 . 24192075

[52] Kim JY, Choi K, Shaker MR, Lee JH, Lee B, Lee E, et al Promotion of cortical neurogenesis from the neural stem cells in the adult mouse subcallosal zone. Stem cells (Dayton, Ohio). 2015 Epub 2015/12/25. 10.1002/stem.2276 . 26701067

[53] Miyamoto N, Maki T, Shindo A, Liang AC, Maeda M, Egawa N, et al Astrocytes Promote Oligodendrogenesis after White Matter Damage via Brain-Derived Neurotrophic Factor. The Journal of neuroscience: the official journal of the Society for Neuroscience. 2015;35(41):14002–8. Epub 2015/10/16. 10.1523/jneurosci.1592-15.2015 26468200 PMC4604233

[54] Preston MA, Macklin WB. Zebrafish as a model to investigate CNS myelination. Glia. 2015;63(2):177–93. Epub 2014/09/30. 10.1002/glia.22755 25263121 PMC4539269

[55] Stewart AM, Braubach O, Spitsbergen J, Gerlai R, Kalueff AV. Zebrafish models for translational neuroscience research: from tank to bedside. Trends in neurosciences. 2014;37(5):264–78. Epub 2014/04/15. 10.1016/j.tins.2014.02.011 24726051 PMC4039217

[56] Fonseka TM, Wen XY, Foster JA, Kennedy SH. Zebrafish models of major depressive disorders. Journal of neuroscience research. 2016;94(1):3–14. Epub 2015/10/11. 10.1002/jnr.23639 . 26452974

[57] Martin-Jimenez R, Campanella M, Russell C. New zebrafish models of neurodegeneration. Current neurology and neuroscience reports. 2015;15(6):33 Epub 2015/04/24. 10.1007/s11910-015-0555-z . 25903297

[58] Babin PJ, Goizet C, Raldua D. Zebrafish models of human motor neuron diseases: advantages and limitations. Progress in neurobiology. 2014;118:36–58. Epub 2014/04/08. 10.1016/j.pneurobio.2014.03.001 . 24705136

[59] Stewart AM, Nguyen M, Wong K, Poudel MK, Kalueff AV. Developing zebrafish models of autism spectrum disorder (ASD). Progress in neuro-psychopharmacology & biological psychiatry. 2014;50:27–36. Epub 2013/12/10. 10.1016/j.pnpbp.2013.11.014 . 24315837

[60] Newman M, Ebrahimie E, Lardelli M. Using the zebrafish model for Alzheimer's disease research. Frontiers in genetics. 2014;5:189 Epub 2014/07/30. 10.3389/fgene.2014.00189 25071820 PMC4075077

[61] Pellegrini E, Mouriec K, Anglade I, Menuet A, Le Page Y, Gueguen MM, et al Identification of aromatase-positive radial glial cells as progenitor cells in the ventricular layer of the forebrain in zebrafish. The Journal of comparative neurology. 2007;501(1):150–67. 10.1002/cne.21222 . 17206614

[62] Zupanc GK. Adult neurogenesis and neuronal regeneration in the central nervous system of teleost fish. Brain, behavior and evolution. 2001;58(5):250–75. 57569. . 11978945 10.1159/000057569

[63] Adolf B, Chapouton P, Lam CS, Topp S, Tannhauser B, Strahle U, et al Conserved and acquired features of adult neurogenesis in the zebrafish telencephalon. Developmental biology. 2006;295(1):278–93. 10.1016/j.ydbio.2006.03.023 . 16828638

[64] Chapouton P, Jagasia R, Bally-Cuif L. Adult neurogenesis in non-mammalian vertebrates. Bioessays. 2007;29(8):745–57. 10.1002/bies.20615 . 17621643

[65] Grandel H, Kaslin J, Ganz J, Wenzel I, Brand M. Neural stem cells and neurogenesis in the adult zebrafish brain: origin, proliferation dynamics, migration and cell fate. Developmental biology. 2006;295(1):263–77. Epub 2006/05/10. 10.1016/j.ydbio.2006.03.040 . 16682018

[66] Barbosa JS, Sanchez-Gonzalez R, Di Giaimo R, Baumgart EV, Theis FJ, Gotz M, et al Neurodevelopment. Live imaging of adult neural stem cell behavior in the intact and injured zebrafish brain. Science (New York, NY). 2015;348(6236):789–93. Epub 2015/05/16. 10.1126/science.aaa2729 . 25977550

[67] Skaggs K, Goldman D, Parent JM. Excitotoxic brain injury in adult zebrafish stimulates neurogenesis and long-distance neuronal integration. Glia. 2014 Epub 2014/07/22. 10.1002/glia.22726 . 25043622 PMC4205181

[68] Diotel N, Vaillant C, Gabbero C, Mironov S, Fostier A, Gueguen MM, et al Effects of estradiol in adult neurogenesis and brain repair in zebrafish. Hormones and behavior. 2013;63(2):193–207. 10.1016/j.yhbeh.2012.04.003 . 22521210

[69] Ganz J, Brand M. Adult Neurogenesis in Fish. Cold Spring Harbor perspectives in biology. 2016 Epub 2016/01/10. 10.1101/cshperspect.a019018 . 26747664 PMC4930922

[70] Menuet A, Pellegrini E, Brion F, Gueguen MM, Anglade I, Pakdel F, et al Expression and estrogen-dependent regulation of the zebrafish brain aromatase gene. The Journal of comparative neurology. 2005;485(4):304–20. 10.1002/cne.20497 . 15803511

[71] Diotel N, Vaillant C, Gueguen MM, Mironov S, Anglade I, Servili A, et al Cxcr4 and Cxcl12 expression in radial glial cells of the brain of adult zebrafish. The Journal of comparative neurology. 2010;518(24):4855–76. 10.1002/cne.22492 . 21031556

[72] Kroehne V, Freudenreich D, Hans S, Kaslin J, Brand M. Regeneration of the adult zebrafish brain from neurogenic radial glia-type progenitors. Development (Cambridge, England). 2011;138(22):4831–41. Epub 2011/10/19. 10.1242/dev.072587 . 22007133

[73] März M, Chapouton P, Diotel N, Vaillant C, Hesl B, Takamiya M, et al Heterogeneity in progenitor cell subtypes in the ventricular zone of the zebrafish adult telencephalon. Glia. 2010;58(7):870–88. 10.1002/glia.20971 . 20155821

[74] Rupp B, Wullimann MF, Reichert H. The zebrafish brain: a neuroanatomical comparison with the goldfish. Anatomy and embryology. 1996;194(2):187–203. . 8827327 10.1007/BF00195012

[75] Mueller T, Wullimann MF. Atlas of Early Zebrafish Brain Development: A Tool for Molecular. American journal of physiology Regulatory, integrative and comparative physiology. 2005:183p.

[76] Marz M, Chapouton P, Diotel N, Vaillant C, Hesl B, Takamiya M, et al Heterogeneity in progenitor cell subtypes in the ventricular zone of the zebrafish adult telencephalon. Glia. 2010;58(7):870–88. Epub 2010/02/16. 10.1002/glia.20971 . 20155821

[77] Coumailleau P, Kah O. Cyp19a1 (aromatase) expression in the Xenopus brain at different developmental stages. Journal of neuroendocrinology. 2014;26(4):226–36. Epub 2014/03/13. 10.1111/jne.12142 24612124 PMC4238815

[78] Coumailleau P, Kah O. Expression of the cyp19a1 gene in the adult brain of Xenopus is neuronal and not sexually dimorphic. General and comparative endocrinology. 2015;221:203–12. Epub 2015/08/11. 10.1016/j.ygcen.2015.08.008 . 26255686

[79] Hill RA. Interaction of sex steroid hormones and brain-derived neurotrophic factor-tyrosine kinase B signalling: relevance to schizophrenia and depression. Journal of neuroendocrinology. 2012;24(12):1553–61. Epub 2012/08/01. 10.1111/j.1365-2826.2012.02365.x . 22845879

[80] Carbone DL, Handa RJ. Sex and stress hormone influences on the expression and activity of brain-derived neurotrophic factor. Neuroscience. 2013;239:295–303. Epub 2012/12/06. 10.1016/j.neuroscience.2012.10.073 23211562 PMC3609934

[81] Hill RA, Wu YW, Kwek P, van den Buuse M. Modulatory effects of sex steroid hormones on brain-derived neurotrophic factor-tyrosine kinase B expression during adolescent development in C57Bl/6 mice. Journal of neuroendocrinology. 2012;24(5):774–88. Epub 2012/01/10. 10.1111/j.1365-2826.2012.02277.x . 22221196

[82] Forlano PM, Deitcher DL, Myers DA, Bass AH. Anatomical distribution and cellular basis for high levels of aromatase activity in the brain of teleost fish: aromatase enzyme and mRNA expression identify glia as source. The Journal of neuroscience: the official journal of the Society for Neuroscience. 2001;21(22):8943–55. . 11698605 10.1523/JNEUROSCI.21-22-08943.2001 PMC6762278

[83] Diotel N, Le Page Y, Mouriec K, Tong SK, Pellegrini E, Vaillant C, et al Aromatase in the brain of teleost fish: expression, regulation and putative functions. Frontiers in neuroendocrinology. 2010;31(2):172–92. 10.1016/j.yfrne.2010.01.003 . 20116395

[84] Diotel N, Vaillant C, Kah O, Pellegrini E. Mapping of brain lipid binding protein (Blbp) in the brain of adult zebrafish, co-expression with aromatase B and links with proliferation. Gene expression patterns: GEP. 2015 Epub 2015/11/28. 10.1016/j.gep.2015.11.003 . 26611722

[85] Tokumine J, Kakinohana O, Cizkova D, Smith DW, Marsala M. Changes in spinal GDNF, BDNF, and NT-3 expression after transient spinal cord ischemia in the rat. Journal of neuroscience research. 2003;74(4):552–61. Epub 2003/11/05. 10.1002/jnr.10760 . 14598299

[86] Burbach GJ, Hellweg R, Haas CA, Del Turco D, Deicke U, Abramowski D, et al Induction of brain-derived neurotrophic factor in plaque-associated glial cells of aged APP23 transgenic mice. The Journal of neuroscience: the official journal of the Society for Neuroscience. 2004;24(10):2421–30. Epub 2004/03/12. 10.1523/jneurosci.5599-03.2004 . 15014117 PMC6729483

[87] Tang YP, Wade J. Sex and age differences in brain-derived neurotrophic factor and vimentin in the zebra finch song system: Relationships to newly generated cells. The Journal of comparative neurology. 2016;524(5):1081–96. Epub 2015/09/12. 10.1002/cne.23893 ; PubMed Central PMCID: PMCPmc4731248. 26355496 PMC4731248

[88] Kim CH, Ueshima E, Muraoka O, Tanaka H, Yeo SY, Huh TL, et al Zebrafish elav/HuC homologue as a very early neuronal marker. Neuroscience letters. 1996;216(2):109–12. Epub 1996/09/27. . 8904795 10.1016/0304-3940(96)13021-4

[89] Janke C, Kneussel M. Tubulin post-translational modifications: encoding functions on the neuronal microtubule cytoskeleton. Trends in neurosciences. 2010;33(8):362–72. Epub 2010/06/15. 10.1016/j.tins.2010.05.001 . 20541813

[90] Ilieva M, Della Vedova P, Hansen O, Dufva M. Tracking neuronal marker expression inside living differentiating cells using molecular beacons. Frontiers in cellular neuroscience. 2013;7:266 Epub 2014/01/17. 10.3389/fncel.2013.00266 ; PubMed Central PMCID: PMCPmc3883158. 24431988 PMC3883158

[91] Nieuwenhuys R. The structural organization of the forebrain: a commentary on the papers presented at the 20th Annual Karger Workshop 'Forebrain evolution in fishes'. Brain, behavior and evolution. 2009;74(1):77–85. Epub 2009/09/05. 10.1159/000229014 . 19729897

[92] Northcutt RG. Do teleost fishes possess a homolog of mammalian isocortex? Brain, behavior and evolution. 2011;78(2):136–8. Epub 2011/09/29. 10.1159/000330830 . 21952091

[93] Mueller T, Dong Z, Berberoglu MA, Guo S. The dorsal pallium in zebrafish, Danio rerio (Cyprinidae, Teleostei). Brain research. 2011;1381:95–105. Epub 2011/01/12. 10.1016/j.brainres.2010.12.089 21219890 PMC3052766

[94] Zhu B, Pennack JA, McQuilton P, Forero MG, Mizuguchi K, Sutcliffe B, et al Drosophila neurotrophins reveal a common mechanism for nervous system formation. PLoS biology. 2008;6(11):e284 Epub 2008/11/21. 10.1371/journal.pbio.0060284 19018662 PMC2586362

