# Supplementary figures and images for: BDNF Expression in Larval and Adult Zebrafish Brain: Distribution and Cell Identification
Source: PLoS One. 2016 Jun 23;11(6):e0158057. doi: 10.1371/journal.pone.0158057 (PMC4918975; doi:10.1371/journal.pone.0158057)

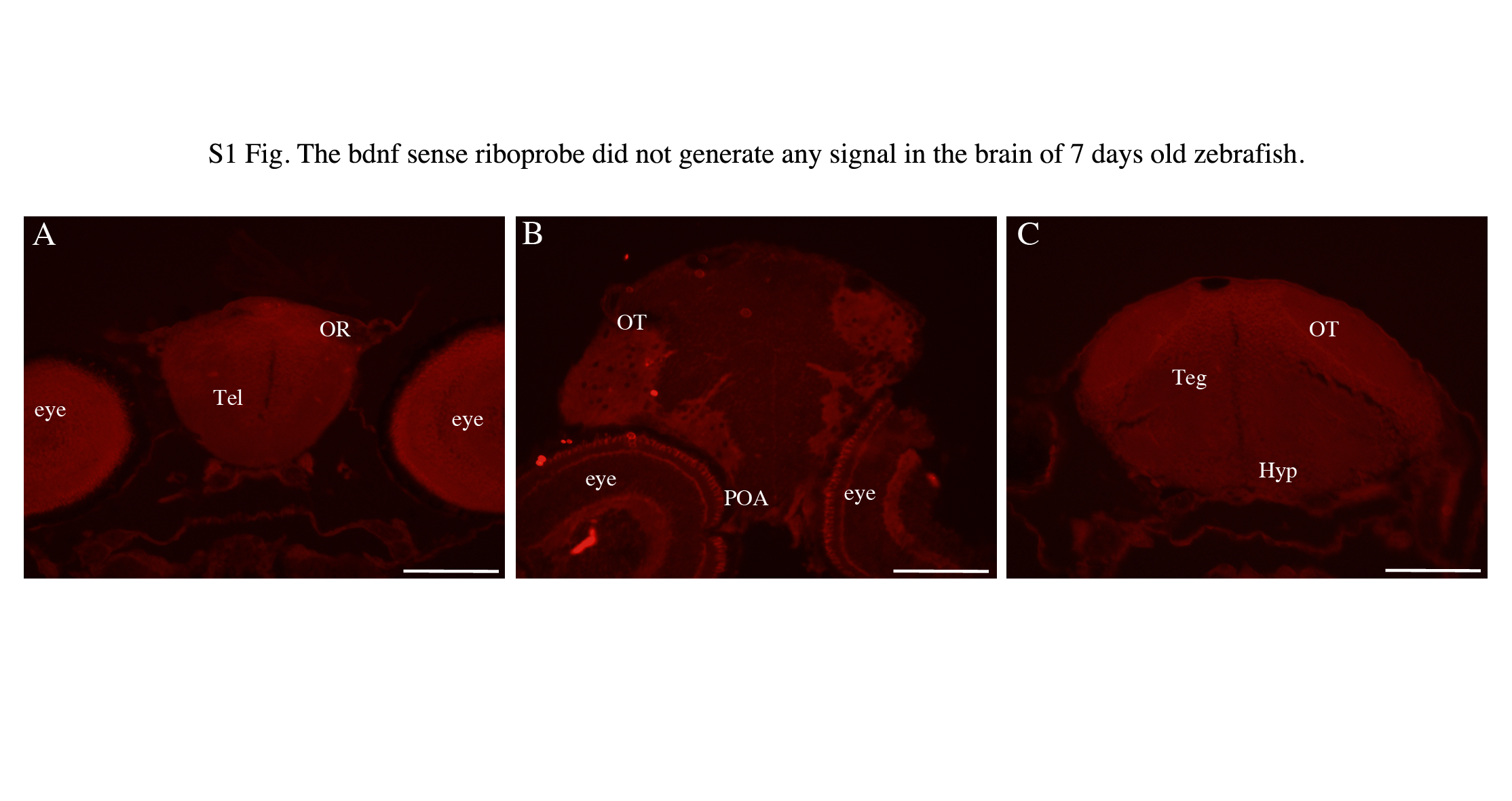

Supplement: S1 Fig — Telencephalon (A), Preoptic area (B), Optic Tectum (B and C). Hyp: hypothalamus; POA: preoptic area; OR: olfactory rosettes; OT: optic tectum. Teg: tegmentum; Tel: telencephalon. Scale bar: 120 μm in A and C. Scale bar: 60 μm in B. (TIF) [file pone.0158057.s001.tif]

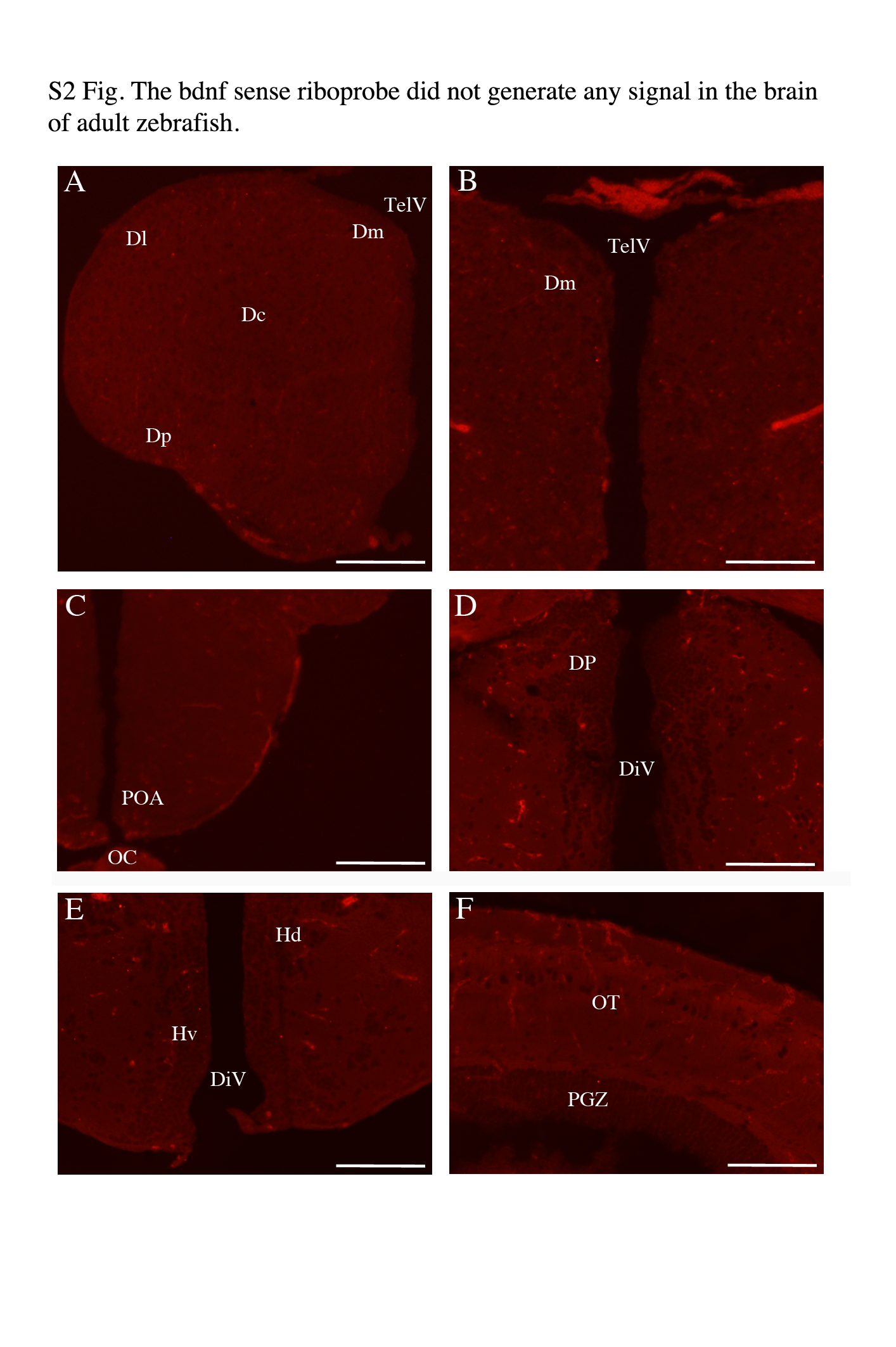

Supplement: S2 Fig — Telencephalon (A and B), Preoptic area (C), Thalamus (D), Hypothalamus (E) and Optic Tectum (F). Dc: central zone of the dorsal telencephalon; DiV: diencephalic ventricle; Dl: lateral zone of the dorsal telencephalon; Dm: medial zone of the dorsal telencephalon; Dp: posterior zone of the dorsal telencephalon; DP: dorsal thalamic nucleus; Hd: dorsal zone of the periventricular hypothalamus; Hv: ventral zone of the periventricular hypothalamus; OC: optic chiasma; OT: optic tectum; PGZ: periventricular gray zone of the optic tectum; POA: preoptic area. Scale bar: 120 μm in A, C. Scale bar: 60 μm in B, D, E, F. (TIF) [file pone.0158057.s002.tif]
